# 16S rRNA Gene-targeted TTGE in Determining Diversity of Gut Microbiota during Acute Diarrhoea and Convalescence

**DOI:** 10.3329/jhpn.v30i3.12287

**Published:** 2012-09

**Authors:** Shirajum Monira, Syeda Antara Shabnam, Nur Haque Alam, Hubert Ph. Endtz, Alejandro Cravioto, Munirul Alam

**Affiliations:** icddr,b, GPO Box 128, Dhaka 1000, Bangladesh

**Keywords:** Child, Convalescence, Diarrhoea, Diarrhoea, Acute, Gut, Microbiota, 16S rRNA gene, Bangladesh

## Abstract

The human gut microbiota play a vital role in health and nutrition but are greatly modified during severe diarrhoea due to purging and pathogenic colonization. To understand the extent of loss during and after diarrhoea, faecal samples collected from children (n=21) suffering from acute diarrhoea and from their healthy siblings (n=9) were analyzed by 16S rRNA gene-targeted universal primer polymerase chain reaction (PCR), followed by temporal temperature gradient gel electrophoresis (TTGE). The gut microbiota decreased significantly as indicated by the number of TTGE bands at day 0 of acute diarrhoea [patients vs healthy siblings: 11±0.9 vs 21.8±1.1 (mean±standard error), p<0.01]. The number of bands showed a steady increase from day 1 to day 7; however, it remained significantly less than that in healthy siblings (15±0.9, p<0.01). These results suggest that appropriate therapeutic and post-diarrhoeal nutritional intervention might be beneficial for the early microbial restoration and recovery.

## INTRODUCTION

The human gastrointestinal tract consists of an extremely complex ecosystem of a diverse microbiota, which is a positive health asset for humans and plays a vital role in the healthy functioning of their human hosts. The gut habitat is highly anoxic, and anaerobic bacteria outnumber the aerobic bacteria by a factor between 10^3^ and 10^5^ (1). Evidence suggests that these commensal microorganisms have important metabolic roles, trophic functions, and protective effects. Moreover, these microbiota exhibit a series of important enzyme activities (2). During the onset of diarrhoea, the anaerobic environment is disrupted due to the high rate of purging, and the commensal microbiota are expelled and replaced by aerobic and pathogenic bacteria. Previous studies have examined the gastrointestinal microbiota in acute cholera (3,4) and in acute diarrhoea cases (5-7), using conventional culture techniques, microscopy, and biochemical tests, showing that the faecal anaerobic bacteria were significantly reduced in number during acute disease leading to aerobic bacteria to increase several folds. The major anaerobic bacterial group, including *Bacteroides, Clostridium, Bifidobacterium, Lactobacillus,* and *Eubacterium,* was found 3-4 times lower in acute cholera and diarrhoea patients. Therefore, the restoration of appropriate commensal gut microbiota, which is the key to recovery following acute diarrhoea, is very important.

The predominant species of human intestinal microbiota are non-culturable because of their obligate anaerobic and extremely oxygen-sensitive nature. Besides, due to the complex nutritional requirements and lack of beneficial effects that they receive from complex host-microbe and microbe-microbe interaction, recent estimates of culturability of bacteria range from only 15% to 58% in the laboratory even when excellent bacteriological culture methods are used (8,9). The metagenomic technique such as temporal temperature gradient gel electrophoresis (TTGE) is an excellent new tool for the analysis of the gut microbiota diversity without depending on the culture method. In TTGE, the variable regions of the 16S rRNA gene are amplified using universal primer PCR, and the amplified DNAs are then separated based on sequence specificity to determine the microbial diversity in the human gut (10-12). Currently, the molecular techniques are being commonly employed to determine the community dynamics in the diverse ecosystems, such as biofilms (13), soils (14), ocean depths (15), hot springs (16), fermented foods (17), and the gut of humans and other animals (18,19).

Many factors, such as age, geographic locations, diets, pH, and bile acid, including intestinal infections, determine the nature and composition of resident bacterial populations in the colon (20). The designing of an appropriate therapeutic intervention involves a clear understanding of the composition of microbiota and their metabolic functions in the gut. In acute diarrhoea, the gut of patients is washed out due to purging and frequent loss of loose stools. As a result, the gut commensal microbiota are likely to be less abundant than those of age-matched healthy children. The aim of the study was to analyze faecal samples of children to determine the extent of loss of gut bacteria during acute diarrhoea and convalescence, using a culture-independent molecular tool—TTGE.

## MATERIALS AND METHODS

### Patients

The study was conducted among children suffering from diarrhoea admitted to an ongoing clinical study of icddr,b during August 2008–May 2009. The children were aged 7-18 months (median 12 months). Faecal samples were collected from 21 children at day 0 (after enrollment and before any medication), at day 1 (after 24 hours), day 2, day 3, and day 7. As a standard management, they received glucose-based oral rehydration solution for rehydration and also received the usual antibiotic therapy, i.e. a parenteral ampicillin dose of 100 mg/kg in four to two equally-divided doses for five days. Faecal samples were also collected once from nine non-diarrhoeal children aged 12-18 months (median 12 months) from siblings of the patients to compare with diarrhoeal children. These children resided at their homes, did not present with diarrhoea, and did not take antibiotics during the last two months.

### Extraction and purification of total DNA

DNA was extracted from the faecal samples following the method of Magne *et al.* (10). One mL of watery stool was centrifuged for 10 minutes at 15,000 g, and the pellet was used for further processing. For semi-solid and solid stools, 125 mg (wet weight) was suspended in 625 µL breaking buffer [0.8 mol/L guanidinium isothiocyanate, 4% N-Lauroylsarcosine, 20 mmol/L Tris (pH 8.0), and 80 mmol/L sodium phosphate buffer (pH 8.0)] and incubated for one hour at 70 °C. Afterwards, 750 µL glass beads of 0.1 mm in diameter (Sigma, St Louis, MO) and 15 mg of polyvinylpolypyrrolidone were added. Bacterial cells were lysed in a vortex mixer at a high speed (10 cycles consisting of one minute of vortexing and one minute of storage in ice). The mixture was centrifuged at 20,000 g for three minutes at 4 °C. After recovery of the supernatant, the pellet was washed three times with 200 µL of TENP [50 mm Tris-HCl (pH 8.0), 20 mm of EDTA (pH 8.0), 100 mm of NaCl, and 1% (w/v) polyvinylpolypyrrolidone]. The four supernatants obtained were pooled. Nucleic acids were extracted with one volume of phenol. The aqueous phase was washed twice using chloroform-isoamyl-alcohol (24:1). DNA was precipitated using 100% isopropanol, and the pellet was washed with 70% v/v isopropanol, dried, and resuspended in 50-100 µL of sterile water and stored at −20 °C. The amount and the integrity of DNA were estimated using 1% (w/v) agarose gel electrophoresis containing ethidium bromide (1 mg/mL) in 1 x TBE (Tris Borate EDTA).

### Polymerase chain reaction/temporal temperature gradient gel electrophoresis

Forward primer S-D-Bact-339-a-S-20 (5’ CTC CTA CGG GAGGCAGCAGT 3’) and reverse primer S-D-Bact-788-a-A-19 (5’ GGA CTA CCA GGG TAT CTA A 3’) were used for amplifying the variable region 3 and 4 of the bacterial 16S rRNA genes in PCR (10). A GC-rich sequence (5’ CCC CCC CCC CCC CGC CCC CCG CCC CCC GCC CCC GCC GCC C 3’) was added to the 5’ end of the reverse primer to prevent the two DNA strands from dissociating completely during TTGE. Each reaction mixture contained 100 ng faecal DNA, 0.5U of Taq DNA polymerase (Takara Bio Inc. Seta 3-4-1, Otsu, Shiga, 520-2193, Japan), 1xreaction buffer, 2.5 mmol/L of MgCl_2_, 0.8 mmol/L of deoxyribonucleotides, and 0.4 mmol/L of primer in a final volume of 20 µL (10). PCR amplifications were performed under the following conditions: initial DNA denaturation and enzyme activation at 94 °C for 10 minutes, then 30 cycles consisting of denaturation (1 minute at 97 °C), annealing (1 minute at 55 °C), elongation (1.5 minutes at 72 °C), and a final elongation at 72 °C for 10 minutes. Their concentration and size (~467 bp) were estimated on a 1% agarose gel containing ethidium bromide (1 mg/mL) in 1 x TBE. The Dcode universal mutation detection system (Bio-Rad, Hercules, CA) was used for sequence-specific separation of amplicons. These amplicons were loaded in the 1 mm polyacrylamide gel consisting of 8% (vol/vol) polyacrylamide (acrylamide:bis acrylamide:: 37.5:1), 7 mol/L urea with 1.25xTAE (40 mmol/L Tris, 20 mmol/L acetate, 1 mmol/L EDTA, pH 8.0) as the electrophoresis buffer. A pre-run of 15 minutes at a constant voltage of 20 V preceded a run at 65 V. The temperature of the gel system was programmed to increase by 0.2 °C per hour from 66 °C to 70 °C. Additionally, similarly-obtained PCR products from known bacterial strains were loaded to allow standardization of band migration and gel curvature among different gels. This ladder consisted of DNA of the following organisms listed in migration order: *Bacteroides* sp., *Vibrio cholerae*, *Enterococcus faecium*, *Staphylococcus epidermidis*, *Escherichia coli*, and *Bifidobacterium longum* (10). Gels were stained with SYBR green I solution (Sigma, St Louis, MO) for 20 minutes, observed under ultraviolet light, and scanned using Gel Doc 2000 (Bio-Rad Inc, Hercules, CA).

### Analysis of TTGE profiles

The TTGE patterns were analyzed using the Diversity Database 2.1, part of the Discovery Series (Bio-Rad). The previously-described analysis is based on the method of Deplancke *et al.* who developed the original strategy (21). Comparisons of TTGE profiles were performed using the Dice similarity coefficient (Dsc) based entirely on results of band classification (22). The Dsc values were compared based on the presence or absence of bands. The Dice coefficient is defined as follows: Dsc=[2j/(a+b)]x100, where j is the number of common bands between sample A and B, and a and b are the total numbers of bands in sample A and B respectively. Consequently, the distance between two TTGE profiles was calculated as follows: Distance=100-Dsc. The coefficient ranged from 0 (no common bands) to 100 (identical band patterns).

### Statistical analysis

Data for the band numbers and distances for different time periods were calculated as mean±standard error of mean, and Student's *t*-test was used for comparing the significant differences between the mean values.

## RESULTS

**Fig. 1.**
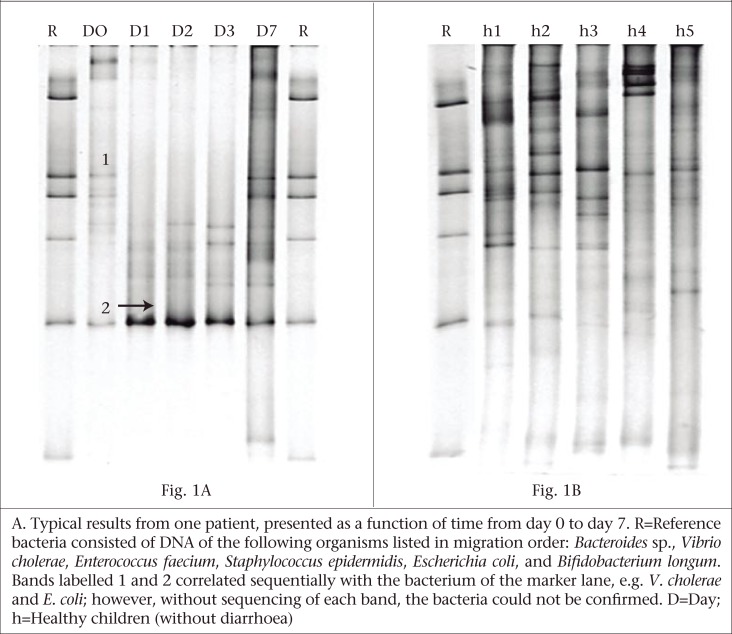
PCR-TTGE analysis of faecal microbiota from children with diarrhoea (A) and children without diarrhoea (B)

A PCR-TTGE analysis of dominant bacteria from the 16S rRNA gene of five different faecal samples from each patient revealed various profiles comprising 3-23 bands. Figure 1A gives one example of a typical result. In a first approximation, each band was assumed to correspond to a different bacterial species because PCR amplicons were obtained from the variable region 3 and 4 of the 16S rRNA gene of mixed microbiota and were separated based on their melting temperature in TTGE gel based on the sequence composition (G+C%) of the amplicons.

At day 0, the number of bands in 14% (3/21) of the children was between 3 and 5; it was between 6 and 9 in 29% (6/21) of the children; and it was between 12 and 15 in the remaining 57% (12/21) of the children. Before any treatment, the average number of bands at day 0 was 10.7±0.9 (mean±standard error) (Table). Compared to day 0, these dominant bacteria were observed to increase significantly (p<0.05) from day 2 until day 7. The results of band profiling of the dominant bacteria in five consecutive faecal samples of children with diarrhoea are shown in the table. After recovery from diarrhoea, the number of bands ranged from 11 to 21 in 20 children at day 7 while one child possessed only six bands. The mean number of bands was significantly higher in children without diarrhoea (21.8±1.1; p<0.01) than in children with diarrhoea at day 7, and the number of bands ranged between 18 and 28 (Fig. 1B).

**Fig. 2.**
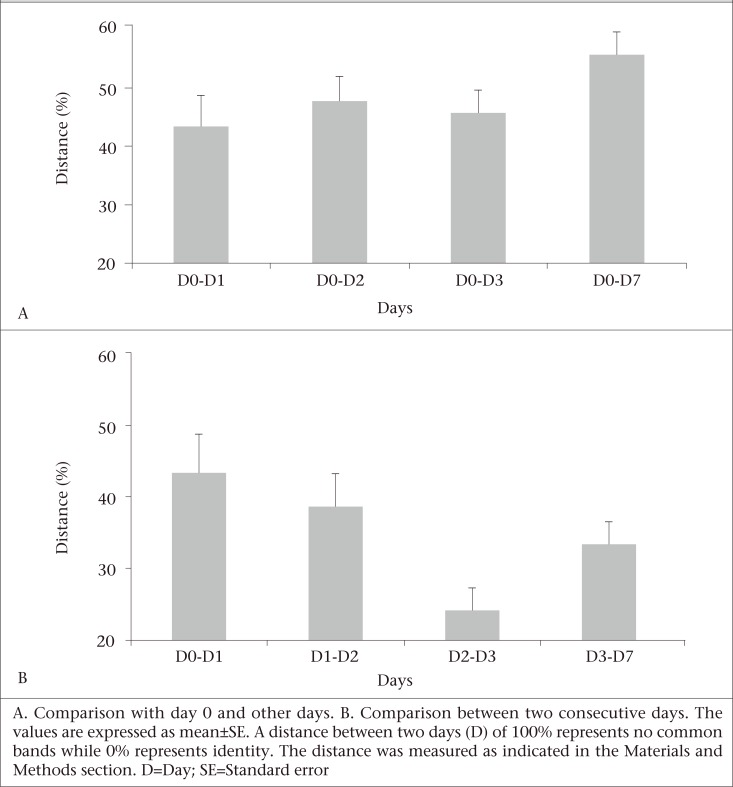
Distances in PCR-TTGE banding patterns of faecal microbiota from children with diarrhoea

The TTGE band profile of each patient was analyzed using Dice similarity coefficient to examine the re-establishment of the microbiota in the gut ecosystem. First, the distance of the band pattern was calculated from the first sample collected between day 0 and day 1, between day 0 and day 2, and so on. Second, the distance between two consecutive samples was calculated between day 0 and day 1, day 1 and day 2, and so on. The mean values of these distances were analyzed to obtain a general picture of the faecal microbiota re-colonization in children with diarrhoea. In the first set of analyses, the TTGE profiles of the patients with acute diarrhoea were compared with the recovering stages (i.e. day 1, day 2, day 3, and day 7), and the average distances were calculated representing the succession of faecal microbiota over these time periods (Fig. 2). In the second set of analyses, the average distances between two consecutive samples were compared to determine the speed of faecal microbiota evolution. The distances in the first analyses were high, indicating that the microbiota were still evolving, and the microbiota profile in the recovery phase was different from that when the patient was experiencing acute diarrhoea. Since the distances in the second comparison (distances between consecutive days) were not very high, these indicate that microbiota during the recovery period were evolving speedily in children leading to a stable microbiota profile.

## DISCUSSION

The study investigated the possible effects of diarrhoea on the diversity of the gut microbiota in children with acute diarrhoea, using a culture-independent metagenomic tool—TTGE. The results presented herein demonstrate a transient reduction in the bacterial diversity that took place after the onset of acute diarrhoea in children. This concurs with results published in recent culture-based studies in which children with diarrhoea were shown to reduce the number of bacterial species (5,7,23). The results of the present study are comparable with a cholera study in which the gut microbiota were analyzed in children suffering from acute cholera using TTGE, and the lower number of bands [7.7±2.6 (mean±standard deviation)] was observed, indicating the lower bacterial diversity in the acute cholera phase (24).

**Table.** Number of PCR-TTGE bands in faecal samples of children with diarrhoea and without diarrhoea*

Band characteristics	Children with diarrhoea (n=21)					Children without diarrhoea (n=9)
	D0	D1	D2	D3	D7	
Number of bands	10.7±0.9	12.2±1.2	14.3±0.8	13.9±1.2	14.9±0.9§	21.8±1.1**

*Values are expressed as mean±SE;

**Comparison between cases with diarrhoea and without diarrhoea at day 7 (p<0.01);

§Comparison between day 0 and day 7 (p<0.01);

SE=Standard error

In the present study, children suffering from diarrhoea received an appropriate rehydration therapy together with antimicrobial therapy and continued to be fed as per the guidelines of the World Health Organization. Although antimicrobials exert a beneficial effect by eliminating pathogenic agents, thereby controlling infections, the oral administration of antimicrobial agents often causes a shift in the intestinal microbial populations, with a decrease in anaerobic bacteria and a concomitant increase in aerobic bacteria (25,26). In the present study, the bacterial diversity in diarrhoea patients, as reflected by the number of different TTGE bands, did not change significantly even after 24 hours of the initiation of antibiotic therapy. However, when the distance values between day 0 and day 1 were analyzed, significant alterations in the bacterial species pattern were observed, suggesting that the antibiotic therapy might have helped the resistant community to selectively flourish at the same time eliminating those microbiota that were sensitive to the drug used, although such shifting was not corroborated in the overall microbiota diversity in the gut as observed at day 0 and day 1.

In our study, no consistency was observed in the banding patterns that occurred in the majority of the patients at day 0 or at day 1, or the following days. The high inter-individual variation in the banding patterns that we observed at day 0 may presumably be due to the differences in their living conditions, health and immune status, and the genetic make-up. However, the band number at day 0 (acute diarrhoea) was enumerated and considered the baseline, and the changes occurring over time in the following days were taken into account as variation. Although a dominant band (bands parallel to *E. coli* in the reference lane) reflecting selective proliferation of a particular species was observed during day 1-3, the overall TTGE profiles for the antibiotic therapy period were more or less static, except that the banding positions were not consistent for every patient that we tested. By contrast, this pattern changed again when the antibiotic therapy ended, and a significant bacterial diversity in terms of numbers and positioning of the dominant bands was observed at day 7. One possible reason for this observation could be that fewer bacterial species could have proliferated in the presence of antibiotics during the clinical period when the sensitive population must have been eliminated/suppressed. This supposition is supported by results of a recent culture-based study in which administration of ampicillin to humans was shown to induce β-lactamase production by various intestinal microorganisms, including Enterobacteriaceae and some members of the *Bacteroides fragilis* group, which allowed simultaneous multiplication of some otherwise ampicillin-sensitive bacteria, such as *Clostridium difficile* (27,28).

In the present study, the TTGE profiles of diarrhoeal and non-diarrhoeal children varied greatly in terms of species richness as revealed by the number of different TTGE bands representing different species. These differences in the numbers of bands were in agreement with the values published in an earlier study (29) which analyzed the influence of host genetics on the predominant bacteria in the faecal microbiota of identical twins, fraternal twins, and unrelated pairs, aged four months to 10 years, showing that the numbers of TTGE bands varied from 8 to 28. The data of the present study may be further substantiated by results of another study in which non-cholera control subjects aged 24-32 months possessed TTGE bands that varied from 20 to 24, showing that these values were almost double when compared with the diversity of microbiota in the gut of children suffering from cholera (24).

### Limitations

This study has limitations as the number of samples was low. Another limitation was the sampling timeframe because one week was not enough for the displaced microbiota to be restored in the gut of post-diarrhoeal children. We believe that follow-up of the patients for an extended period of time would be needed to get a clearer picture of status of the gut microbiota in post-diarrhoeal children. Although the identification of TTGE bands representing different species would be interesting, in the present study, the intensity of the majority of the bands was very low and was not suitable for the purpose of purification of DNA by cutting TTGE bands for sequencing and analyses. Again, the positions of bands varied greatly in day 1 to day 3; so, extensive cloning and sequencing of dominant bands during treatment were also not suitable.

### Conclusions

The results of this study clearly showed that the bacterial diversity of the gut microbiota was significantly reduced during acute diarrhoea and that the diversity did not reach the level of non-diarrhoeal control children after one week. It is presumed that nutritional and appropriate therapeutic intervention during the post-diarrhoeal phase might be beneficial for achieving a higher magnitude of the diversity of the gut microbiota similar to that found in non-diarrhoeal children in a shorter duration.

## ACKNOWLEDGEMENTS

The study was conducted at icddr,b with support from Core Fund. The authors acknowledge the excellent service and assistance of the physicians and nurses at the Dhaka Hospital of icddr,b.
